# Efficacy and safety of denosumab in treatment of osteoporosis in patients with rheumatoid arthritis: a meta-analysis and systematic review

**DOI:** 10.1186/s12891-025-09206-6

**Published:** 2025-10-09

**Authors:** Runyao Zhang, Nannan Kou, Feifei Liu, Hongliang Zhou, Lirong Ren

**Affiliations:** 1 Department of orthopaedics, Guiqian International Hospital, Guiyang, Guizhou Province 550024 P.R. China; 2 Department of Trauma surgery, the Second Affiliated Hospital of Kunming Medical University, Yunnan Province Kunming, 650000 P.R. China; 3 Department of Spinal surgery, The First Affiliated Hospital of Dali University, Yunnan Province Dali, 671000 P.R. China

**Keywords:** Denosumab, Rheumatoid arthritis, Osteoporosis, Efficacy and safety, Meta-analysis and systematic review

## Abstract

**Objective:**

This meta-analysis aims to assess the efficacy and safety of denosumab in treating osteoporosis (OP) in patients with rheumatoid arthritis (RA).

**Methods:**

We conducted a comprehensive search in databases including PubMed, Embase and Cochrane Library for studies on denosumab's effectiveness in treating OP in RA patients from their inception until 2023. Two researchers independently screened the retrieved literature, and the methodological quality of the included studies was evaluated using the Cochrane 5.1 bias risk assessment tool. Statistical analysis was performed using STATA 15.1.

**Results:**

A total of 1441 patients from 9 articles, meeting the specified criteria, were included, with 762 in the experimental group and 679 in the control group. The pooled results suggest that following the treatment of osteoporosis in patients with rheumatoid arthritis, both bone mineral density (BMD) and bone erosion score (BES) exhibit notable increases with denosumab in comparison to biological agent therapy. Moreover, the modified total Sharp score (mTSS), joint space narrowing (JSN), and BES demonstrate significantly higher improvements subsequent to denosumab therapy for osteoporosis in rheumatoid arthritis patients, as opposed to receiving a placebo. However, there is no significant difference between denosumab and placebo treatment for osteoporosis in patients with RA in terms of serious adverse events.

**Conclusion:**

In conclusion, compared to the control group, denosumab treatment significantly increased lumbar spine BMD in RA patients who had not received BPs treatment. Additionally, it reduced joint mTSS, JSN, and BES, alleviated joint space narrowing, and inhibited bone erosion, providing maximal joint protection and maintaining joint function.

**Supplementary Information:**

The online version contains supplementary material available at 10.1186/s12891-025-09206-6.

## Introduction

Rheumatoid arthritis (RA) is a systemic autoimmune disease characterized by chronic synovial joint inflammation, leading to progressive joint destruction, bone and cartilage damage, joint deformities, functional impairment, and eventual disability, significantly impacting the quality of life of affected individuals. RA patients are at a higher risk of osteoporosis and brittle bone fractures compared to normal controls. Glucocorticoids, commonly used in RA treatment, can lead to osteoporosis with long-term use, particularly in elderly female RA patients, resulting in increased susceptibility to brittle bone fractures.

Denosumab is a humanized IgG2 monoclonal antibody with high affinity for the receptor activator for NF-κB ligand (RANKL). Approved by the U.S. FDA in 2009 for postmenopausal osteoporosis in women, denosumab inhibits bone resorption by binding to the RANKL signaling pathway. In 2019, it was approved in China. The binding of RANKL to RANK induces the differentiation, maturation, and activation of osteoclasts, promoting bone absorption. Denosumab, by inhibiting RANKL, suppresses osteoclast bone resorption and has been proven to slow the progression of joint damage and improve osteoporosis. Several randomized controlled trials (RCTs) both domestically and internationally have investigated the use of denosumab in treating osteoporosis in RA patients, and its clinical application is increasing.

Although several randomized controlled trials and observational studies have explored the efficacy of denosumab in osteoporosis and RA separately, high-quality evidence specifically addressing its role in RA patients with concomitant osteoporosis remains limited. Previous studies have often involved small sample sizes, heterogeneous outcome measures, and varying control regimens, making it difficult to draw robust and generalizable conclusions. Moreover, few meta-analyses have systematically compared denosumab with both bisphosphonates and placebo across key structural and functional outcomes. Therefore, this study conducts a comprehensive meta-analysis to evaluate the efficacy and safety of denosumab in treating osteoporosis in RA patients, aiming to fill this important evidence gap and provide stronger evidence-based guidance for clinical decision-making.

## Methods

### Literature inclusion and exclusion criteria

Inclusion criteria:

1. subjects: patients with osteoporosis and rheumatoid arthritis
2. intervention measure: denosumab
3. control: bisphosphonates (BPs) or a placebo (administration, dosage, and frequency not specified)
4. Outcome indicators: Bone mineral density (BMD), modified Sharp score (mTSS), joint space narrowing score (JSN), bone erosion score (BES), and incidence of severe adverse events.
5. Study design: randomized controlled trial (RCT) and observational study

Exclusion criteria:

Duplicate publications; studies for which full text was not available or for which data extraction was not possible; studies using animal studies; reviews and systematic reviews.

### Search strategy

For this meta-analysis, we conducted searches in PubMed, Embase and the Cochrane Library databases from their inception until December 2023. The search terms used were: “denosumab” AND “rheumatoid arthritis” AND “osteoporosis”.

### Literature screening and data extraction

All literature retrieval and data extraction processes were carried out independently by two investigators. Any inconsistencies encountered during the process were addressed through discussion, with the involvement of a third researcher if necessary. The initial step included de-duplication using reference management software. Titles and abstracts were then screened to exclude studies that were clearly irrelevant to the topic. For potentially eligible articles, full texts were obtained and thoroughly assessed to determine final inclusion based on predefined criteria. Extracted data included key study characteristics such as first author, year of publication, study location, sample size, participant demographics (including age and gender), disease course, treatment methods, duration of therapy, and reported outcome measures.

### Literature quality assessment

The quality assessment of cohort studies was independently performed by two reviewers using the Newcastle-Ottawa Scale (NOS). This tool evaluates studies across three domains: selection of participants (4 items, up to 4 points), comparability of study groups (1 item, up to 2 points), and outcome assessment (3 items, up to 3 points), with a Maximum total score of 9. Studies scoring 7 points or higher were considered high quality, while those scoring below 7 were categorized as lower quality. Any discrepancies in scoring were resolved through discussion or, if needed, by consulting a third reviewer. The entire meta-analysis process adhered to the methodological framework outlined in the PRISMA (Preferred Reporting Items for Systematic Reviews and Meta-Analyses) statement.

### Data synthesis and statistical analysis

Statistical analyses were conducted using STATA version 15.1. For continuous outcomes, standardized mean differences (SMDs) with 95% confidence intervals (CIs) were calculated, while odds ratios (ORs) with 95% CIs were used for dichotomous variables. Heterogeneity across studies was assessed using the I² statistic and chi-square test. When heterogeneity was low (I² ≤ 50% and *P* ≥ 0.1), a fixed-effects model was applied. In contrast, when substantial heterogeneity was detected (I² >50% and *P* < 0.1), a sensitivity analysis was performed to explore potential sources of variability. If heterogeneity remained unexplained, a random-effects model was employed; in cases of extreme inconsistency, a descriptive summary was preferred over pooled results. Potential publication bias was evaluated using funnel plot symmetry and Egger’s test.

## Results

### Literature screening results

**Fig. 1:**
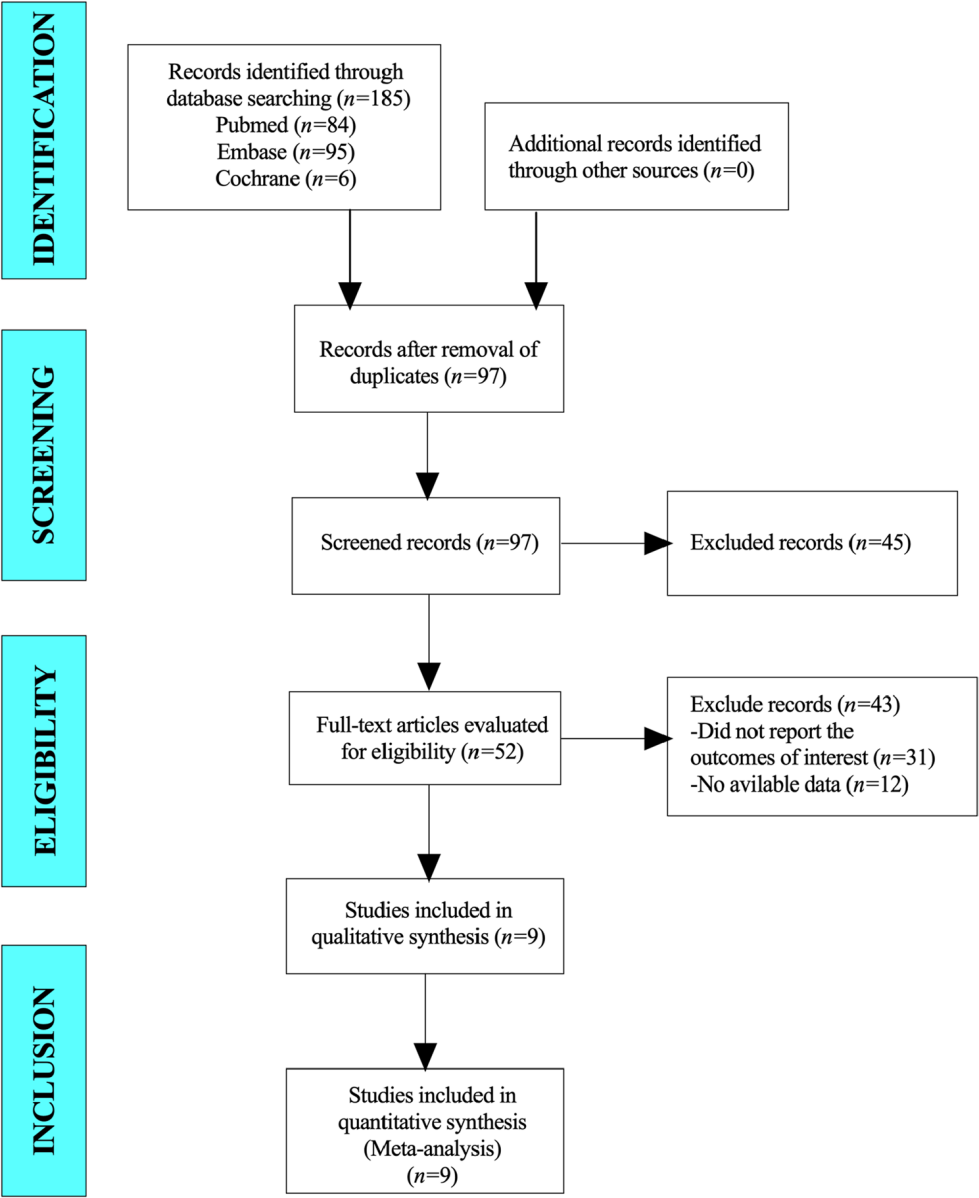
Flow diagram for selection of studies

A total of 185 articles were collected for this study. After excluding duplicate studies, 97 studies were included in this study. A total of 52 articles were identified after reading their titles and abstracts. Finally, 9 studies were included in the meta-analysis (Fig. 1).

### Basic characteristics of included literature

**Fig. 2:**
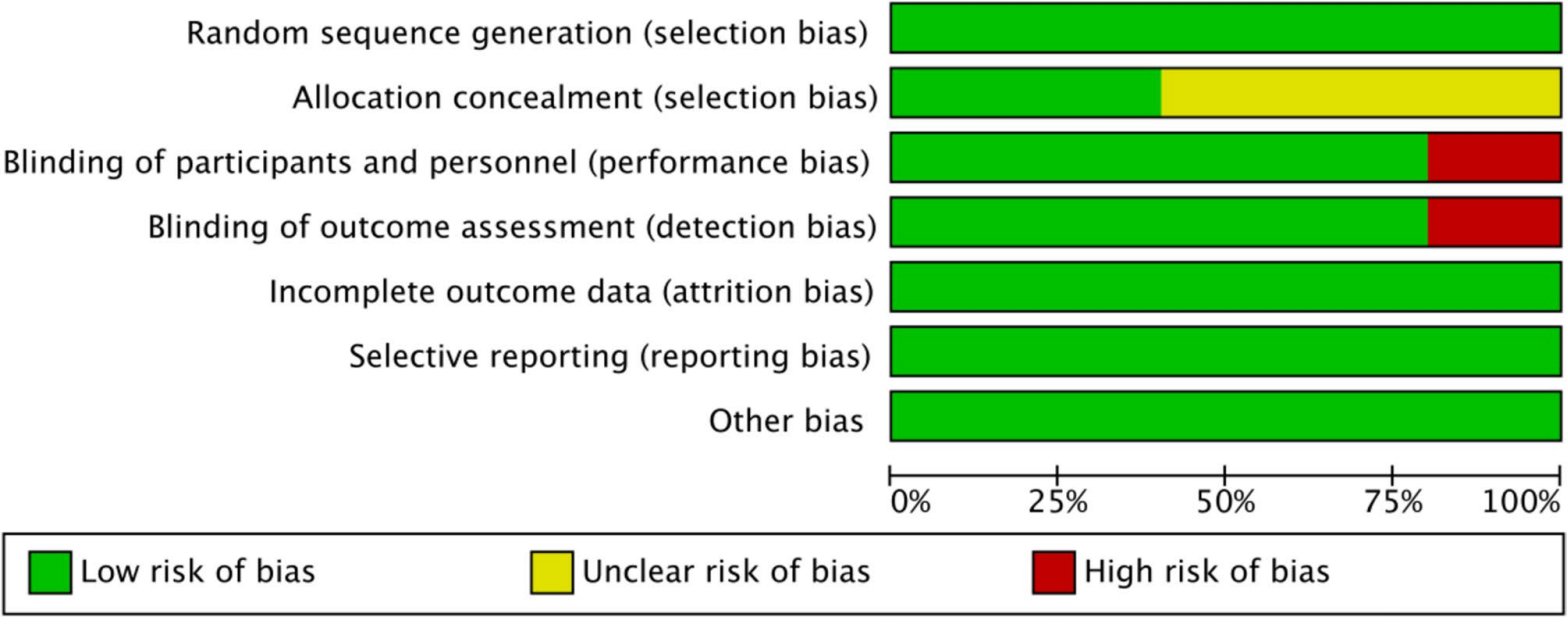
Risk of bias graph

**Fig. 3:**
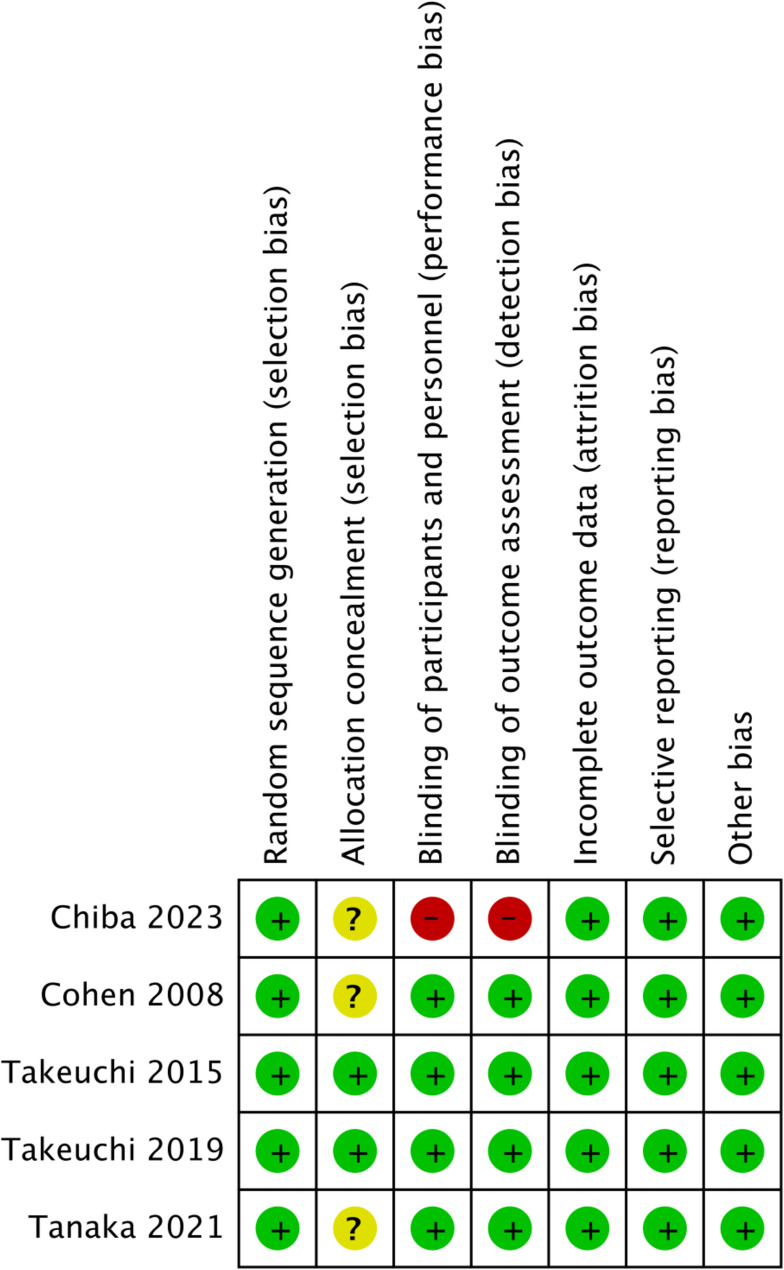
Risk of bias summary

A total of 9 articles were included, comprising 5 RCTs and 4 cohort studies. The basic characteristics of the literature are presented in Table 1. The inclusion involved 1441 patients, with 762 in the experimental group and 679 in the control group. The average age distribution of patients in the experimental group ranges from 54.4 to 74.1, while in the control group, the age of patients ranges from 57.0 to 70.3. The average disease duration of patients in the experimental group ranges from 2.16 to 18.2, while in the control group, it ranges from 2.31 to 18.3. This demonstrates the comparability between the two groups in terms of demographic characteristics. The NOS scores used for quality assessment were all > 7 and met the requirements (Table 1). The Cochrane Bias Risk Assessment tool was employed to evaluate the quality of the RCTs. Of the 5 RCTs, none exhibited incomplete data, selective reporting, or other biases. Three studies did not describe whether to allocation concealment, while only one article did not use blinding (Figs. 2 and 3).

**Table 1:** Baseline characteristics of the included studies

Author	Year	Study design	Country	Sample size		Age		Gender (female/male)		Intervention measure		Disease duration (years)		NOS score
				Experimental group	Control group	Experimental group	Control group	Experimental group	Control group	Experimental group	Control group	Experimental group	Control group	
Cohen	2008	RCT	USA	73	78	57.3 ± 11.4	57.0 ± 11.1	62/16	51/22	Denosumab	Placebo	9.7 ± 8.1	10.5 ± 7.2	/
Takeuchi	2015	RCT	Japan	85	88	54.4 ± 10.6	57.0 ± 10.6	75/20	76/12	Denosumab	Placebo	2.16 ± 1.31	2.31 ± 1.34	/
Hasegawa	2016	Cohort	Japan	40	40	74.1 ± 10.2	70.3 ± 8.1	38/2	37/3	Denosumab	Biological agent	14.1 ± 8.9	12.1 ± 10.4	7
Kinoshita	2016	Cohort	Japan	49	49	70.6 ± 7.6	67.7 ± 10.0	47/2	46/3	Denosumab	BPs	12.4 ± 9.8	13.2 ± 11.4	7
Ebina	2018	Cohort	Japan	30	30	68.5 ± 1.8	67.6 ± 1.8	/	/	Denosumab	BPs	18.2 ± 2.4	18.3 ± 1.9	8
Takeuchi	2019	RCT	Japan	217	218	58.1 ± 12.3	55.8 ± 11.7	168/49	167/51	Denosumab	Placebo	2.20 ± 1.33	2.07 ± 1.30	/
Tanaka	2021	RCT	Japan	191	105	57.2 ± 12.1	54.5 ± 12.6	150/41	81/24	Denosumab	Placebo	2.3 ± 1.3	2.3 ± 1.4	/
Mori	2021	Cohort	Japan	56	50	71.1 ± 8.2	67.6 ± 7.4	/	/	Denosumab	BPs	11.3 ± 5.2	9.7 ± 5.1	7
Chiba	2023	RCT	Japan	21	21	65.6 ± 8.5	65.0 ± 9.2	18/3	20/2	Denosumab	Biological agent	13.5 ± 12.6	12.5 ± 10.8	/

### Meta-analysis results

#### BMD

**Fig. 4:**
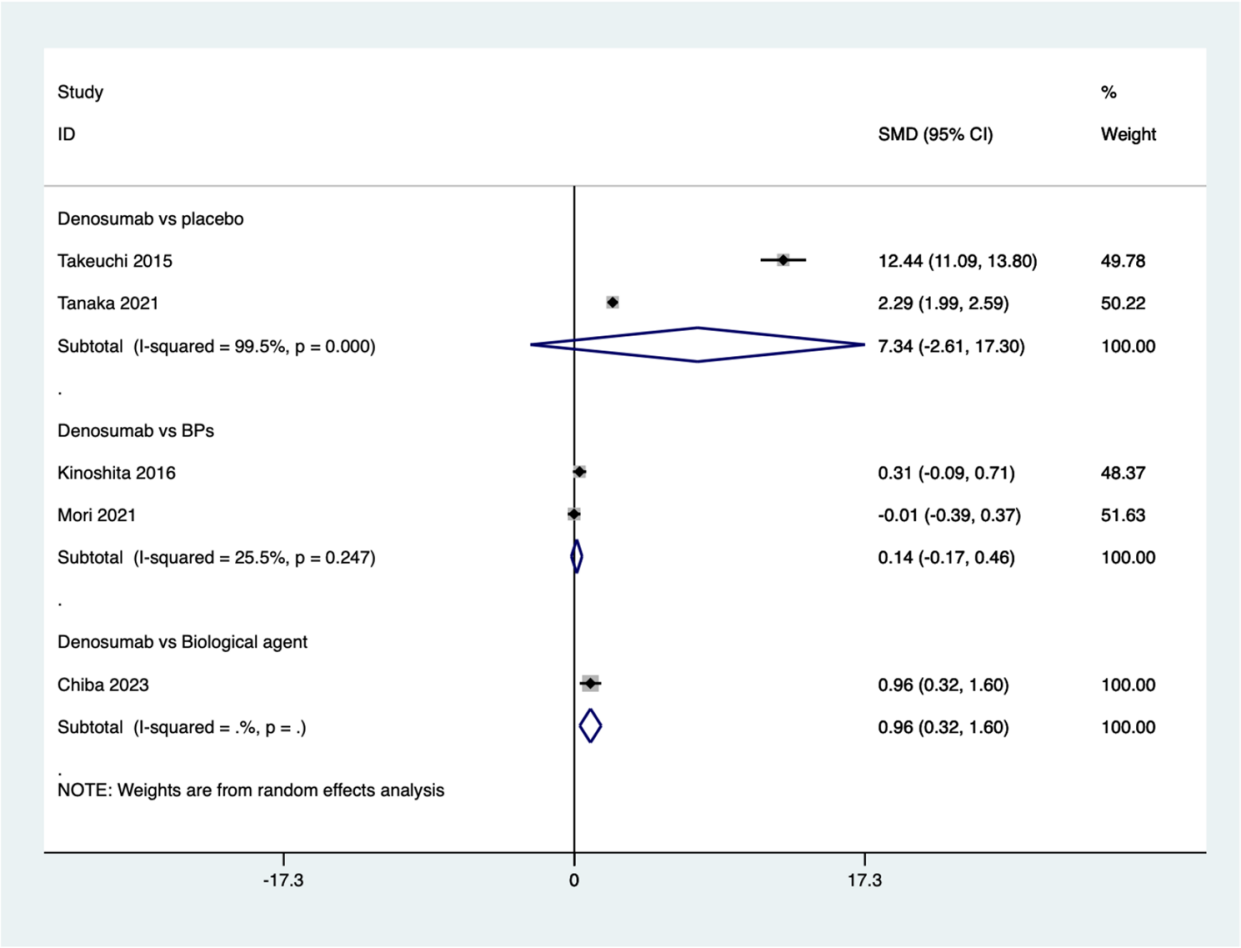
Comparing the differences in BMD between denosumab group and control group in the treatment of osteoporosis in patients with rheumatoid arthritis

Five studies reported the improvement in lumbar spine BMD. The summarized results indicate that after treating osteoporosis in patients with rheumatoid arthritis, the BMD is significantly higher with denosumab compared to biological agent treatment (SMD = 0.96, 95%CI: 0.32–1.60, *P* = 0.003). However, there is no significant difference between denosumab and placebo (SMD = 7.34, 95%CI: −2.61 ~ 17.20, *P* = 0.148; *I*^*2*^ *= 99.5%*, *P* *= 0.000*) or BPs (SMD = 0.14, 95%CI: −0.17 ~ 0.46, *P* = 0.374; *I*^*2*^ *= 25.5%*, *P* *= 0.247*) (Fig. 4).

#### mTSS

**Fig. 5:**
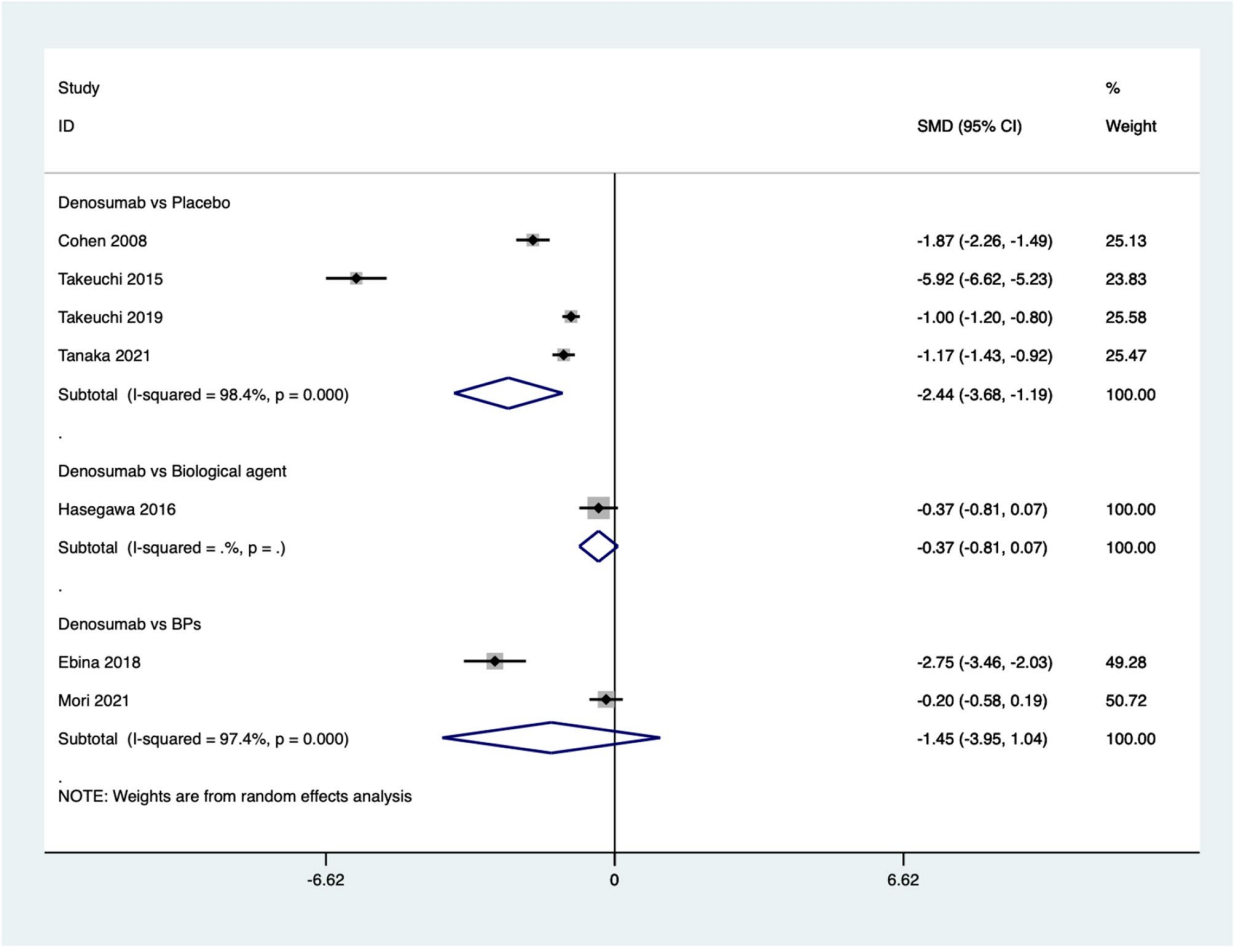
Comparing the differences in mTSS between denosumab group and control group in the treatment of osteoporosis in patients with rheumatoid arthritis

Seven studies reported the improvement in the mTSS. The pooled results indicate that the mTSS after denosumab treatment for osteoporosis in patients with rheumatoid arthritis is significantly higher compared to placebo (SMD=−2.44, 95%CI: −3.68~−1.19, *P* = 0.000; *I*^*2*^ *= 98.4%*, *P* *= 0.000*). However, there is no significant difference between denosumab and biological agents (SMD=−0.37, 95%CI: −0.81 ~ 0.07, *P* = 0.101) or BPs (SMD=−1.45, 95%CI: −3.95 ~ 1.04, *P* = 0.254; *I*^*2*^ *= 97.4%*, *P* *= 0.000*) (Fig. 5).

#### JSN

**Fig. 6:**
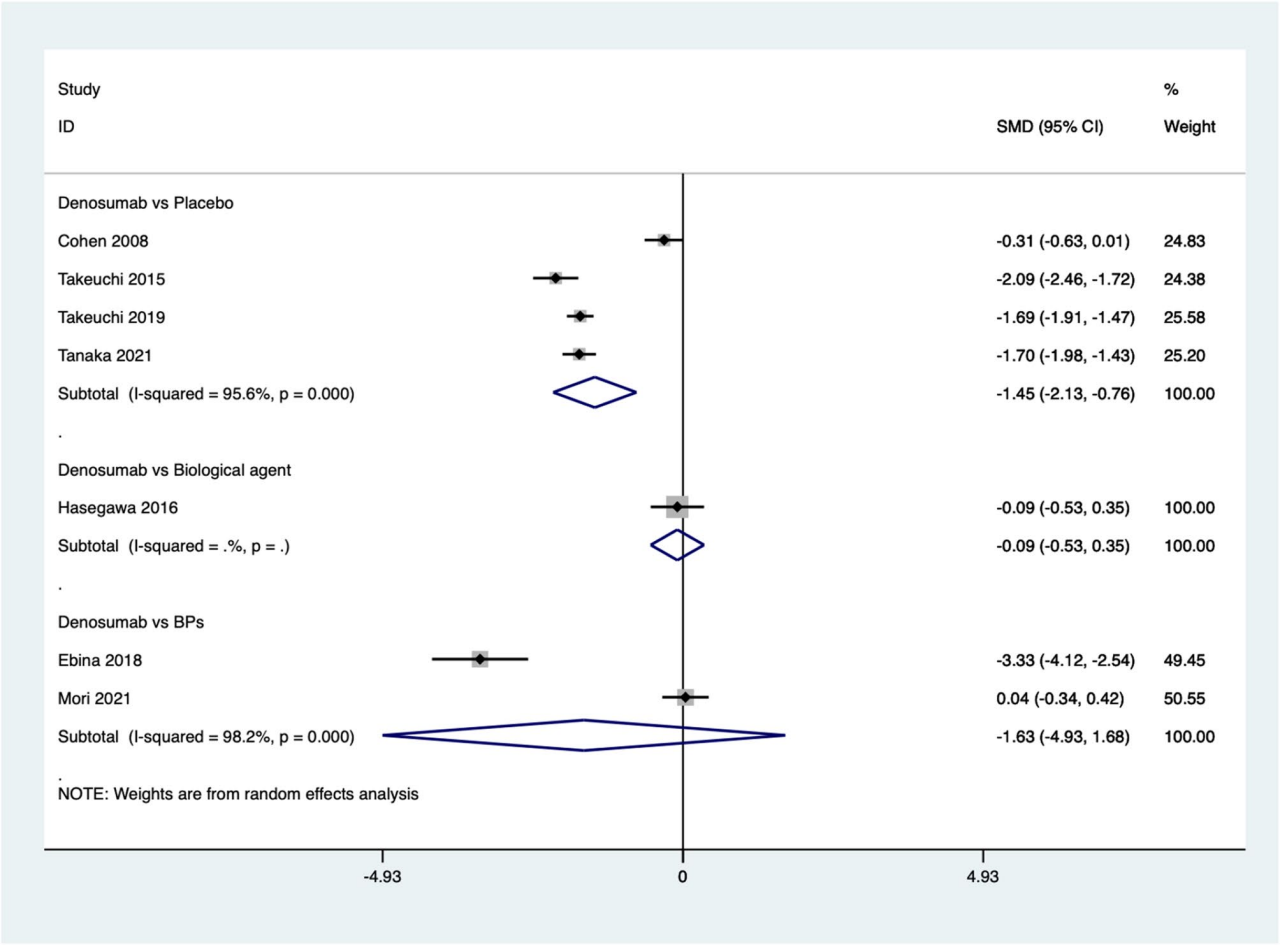
Comparing the differences in JSN between denosumab group and control group in the treatment of osteoporosis in patients with rheumatoid arthritis

Seven studies reported improvement in JSN. The pooled results indicate that the JSN after denosumab treatment for osteoporosis in patients with rheumatoid arthritis is significantly higher compared to placebo (SMD=−1.45, 95%CI: −2.13~−0.76, *P* = 0.000; *I*^*2*^ *= 95.6%*, *P* *= 0.000)*. However, there is no significant difference between denosumab and biological agents (SMD=−0.09, 95%CI: −0.53 ~ 0.35, *P* = 0.681) or BPs (SMD=−1.63, 95%CI: −4.93 ~ 1.68, *P* = 0.335; *I*^*2*^ *= 98.2%*, *P* *= 0.000)* (Fig. 6).

#### BES

**Fig. 7:**
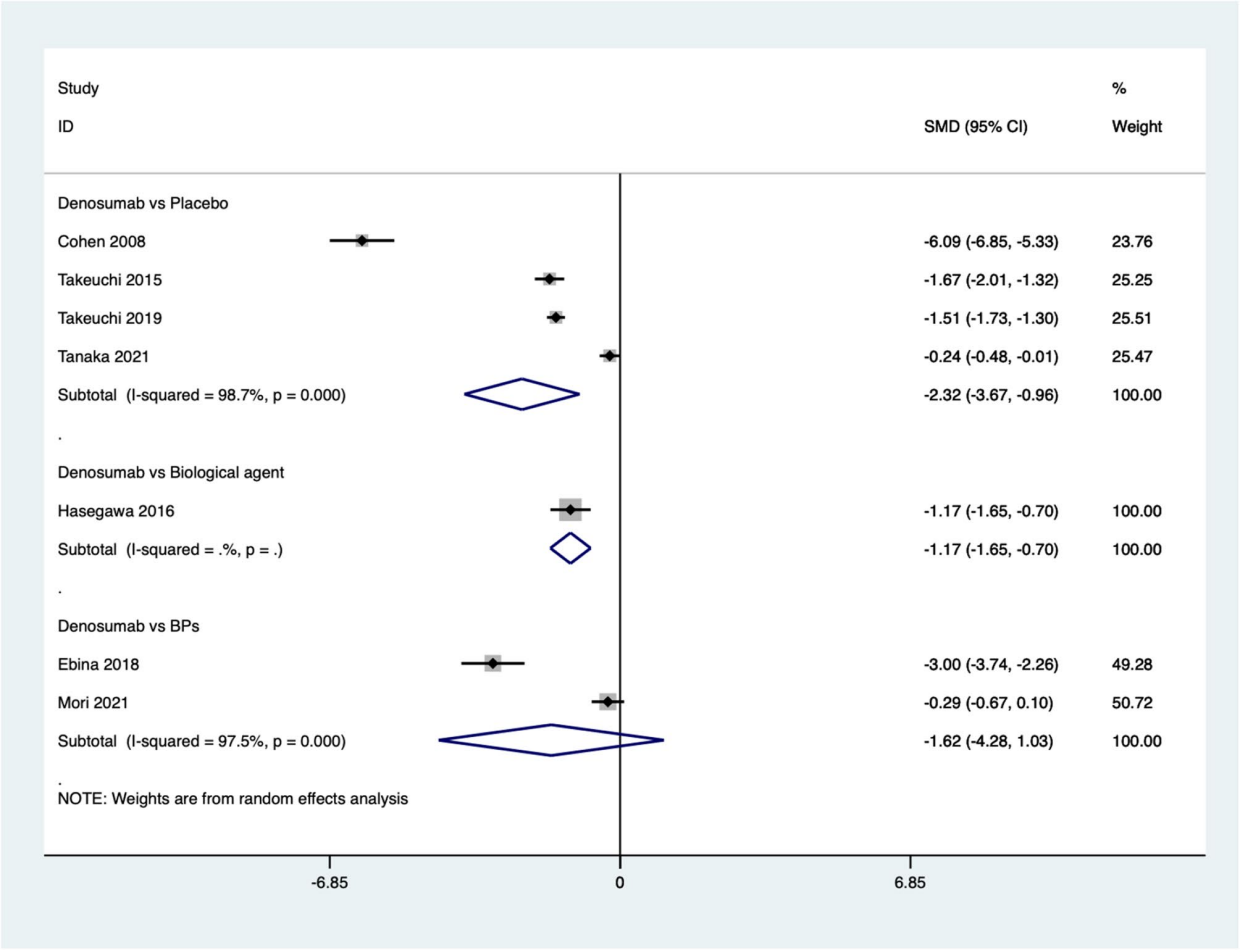
Comparing the differences in BES between denosumab group and control group in the treatment of osteoporosis in patients with rheumatoid arthritis

Seven studies reported improvement in BES. The pooled results indicate that the BES after denosumab treatment for osteoporosis in patients with rheumatoid arthritis is significantly higher compared to placebo (SMD=−2.32, 95%CI: −3.67~−0.96, *P* = 0.001; *I*^*2*^ *= 98.7%*, *P* = 0.000) or biological agents (SMD=−1.17, 95%CI: −1.65~−0.70, *P* = 0.000). However, there is no significant difference between denosumab and BPs (SMD=−1.62, 95%CI: −4.28 ~ 1.03, *P* = 0.231; *I*^*2*^ *= 97.5%*, *P* = 0.000) (Fig. 7).

#### Serious adverse events

**Fig. 8:**
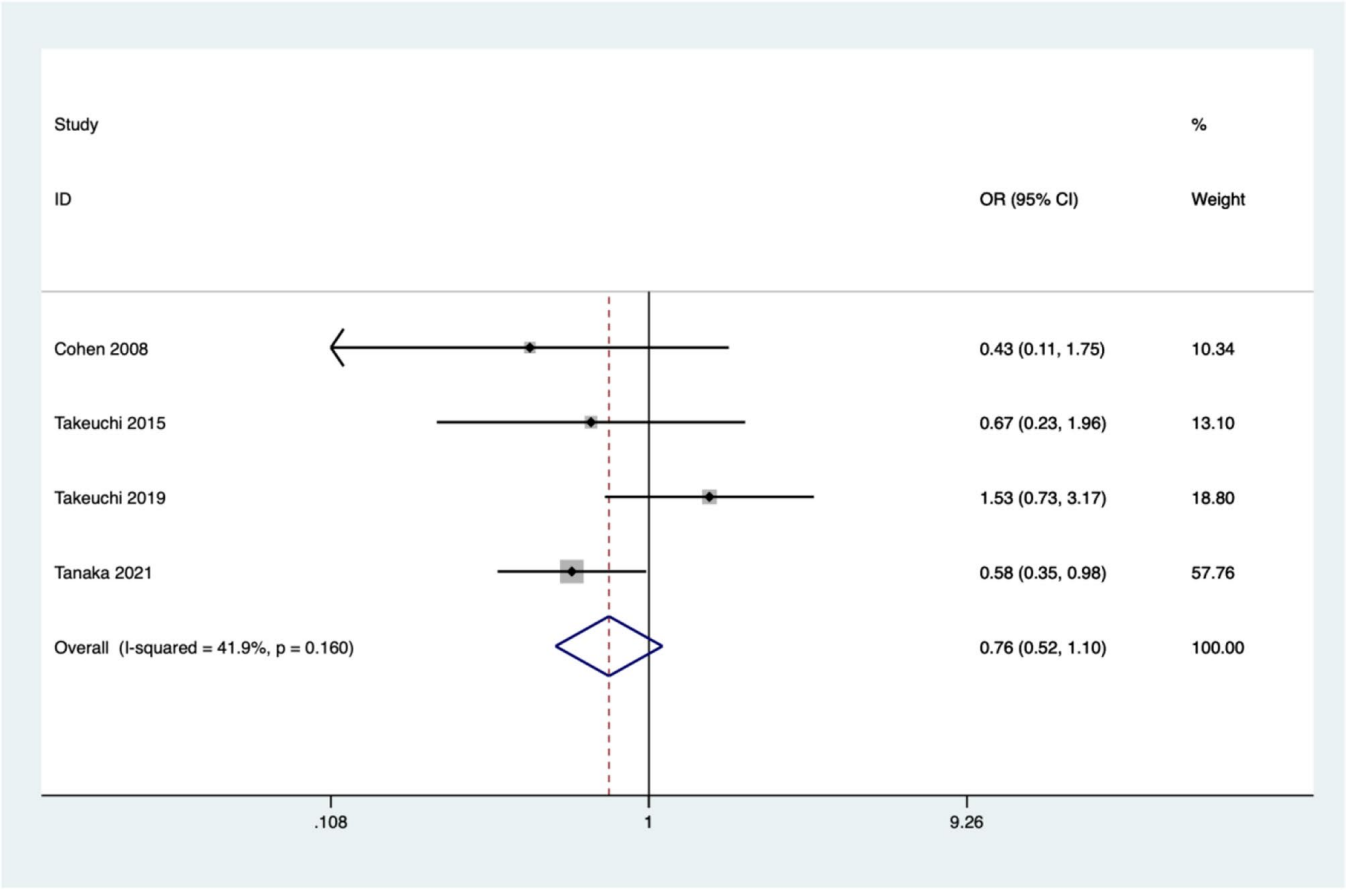
Comparing the differences in serious adverse events between denosumab group and control group in the treatment of osteoporosis in patients with rheumatoid arthritis

Four studies reported differences in adverse events between denosumab and placebo. The summary results indicate that there is no significant difference between denosumab and placebo treatment for osteoporosis in patients with rheumatoid arthritis in terms of serious adverse events (OR = 0.76, 95%CI: 0.52 ~ 1.10, *P* = 0.143; *I*^*2*^ *= 41.9%*, *P* = 0.160) (Fig. 8).

## Discussion

Local and systemic bone loss are major extra-articular complications of rheumatoid arthritis (RA), leading to increased risks of osteoporosis (OP) and fragility fractures. This further damages joint function, reduces quality of life, and shortens life expectancy. It has been reported that about 30% of RA patients have an increased risk of OP, emphasizing the urgent need for effective management of OP and fracture risks in RA patients [22]. Bone mineral density (BMD) measurements play a crucial role in assessing bone loss and preventing fractures [23]. Despite the attention given to OP treatment, current medications, particularly bisphosphonates (BPs), still present limitations. Several new drugs have been developed, with denosumab showing significant potential in treating OP. Since its approval, denosumab has demonstrated strong inhibition of bone resorption, increased BMD, and prevention of fragility fractures, offering new hope for treating OP in RA patients [24].

Numerous foreign studies have conducted clinical trials to assess the effectiveness and safety of denosumab in treating OP in RA patients. A meta-analysis by BOLETO et al. [25] suggested that denosumab is effective in joint damage at 6 and 12 months compared to placebo, alendronate, and biologic disease-modifying antirheumatic drugs (bDMARDs). However, no impact on joint space narrowing was observed. Safety profiles, including adverse events, bone necrosis, or atypical fracture cases, were similar between denosumab and control groups. Additionally, denosumab demonstrated therapeutic efficacy in joint erosion in RA patients at 6 and 12 months, regardless of concurrent biologic agent therapy, with good safety.

There is a lack of reported studies on denosumab for treating OP in RA patients in China, likely due to the relatively recent introduction of the drug to the domestic Market. To further advance clinical treatment for RA patients with OP, this study conducted a meta-analysis of eight relevant foreign publications. The results indicated that denosumab significantly increased lumbar spine BMD after 12 months of treatment, decreased modified total Sharp score (mTSS), joint space narrowing (JSN), and bone erosion score (BES), alleviated joint space stenosis, inhibited bone erosion damage, and Maximally protected the joints. Moreover, the occurrence of general and serious adverse events in the denosumab group was statistically similar to the control group. Although there were three deaths in the denosumab group among 566 patients, the difference was not statistically significant compared to the control group.

From a clinical perspective, our findings suggest that while denosumab significantly improved certain structural outcomes and BMD in RA patients with concomitant osteoporosis, its effects were not consistently superior to those of placebo or bisphosphonates across all measured endpoints. The lack of significant differences in some comparisons may reflect several factors: (1) bisphosphonates remain effective antiresorptive agents and may provide comparable benefits for specific outcomes such as lumbar spine BMD in the short to medium term; (2) placebo groups in some trials were allowed concomitant standard RA therapy, which could attenuate between-group differences; and (3) variability in follow-up duration, baseline disease activity, and prior osteoporosis treatment history may have influenced the observed treatment effects. Clinically, these results highlight the importance of individualized treatment selection. Denosumab may be most relevant for patients at high risk of rapid bone loss or fracture who have contraindications to bisphosphonates, poor adherence to oral regimens, or inadequate response to previous antiresorptive therapy. Conversely, for patients with stable disease activity and preserved bone mass, conventional therapies such as bisphosphonates may remain a reasonable and cost-effective option. These nuances should be considered when integrating denosumab into treatment algorithms for RA-related osteoporosis.

This study has certain limitations. Denosumab has only recently entered the Chinese market, resulting in few randomized controlled trials conducted on the Chinese population, which may affect the applicability of the findings to domestic settings. The number of included studies is relatively small, and nearly all were conducted in Japan, which may limit the generalizability of the results to other geographic regions and ethnic groups. In addition, there was substantial heterogeneity among the included studies in terms of design and population characteristics, including differences in study type, patient demographics, disease duration, baseline bone status, and concomitant therapies, which may have influenced the pooled estimates. Furthermore, the literature search did not include Scopus or Web of Science, which may limit the comprehensiveness of the review; inclusion of these databases in future updates could further enhance the robustness and relevance of the findings.

## Conclusion

In conclusion, compared to the control group, denosumab treatment for 12 months significantly increased lumbar spine BMD in RA patients who had not received BPs treatment. Additionally, it reduced joint mTSS, JSN, and BES, alleviated joint space narrowing, and inhibited bone erosion, providing maximal joint protection and maintaining joint function. Regarding safety, the occurrence of adverse events was statistically similar between the two groups, indicating a high level of safety. However, due to the relatively small number of cases included in this study, further verification through larger sample sizes, long-term treatment, follow-up, and more comprehensive analysis of outcome indicators and adverse reactions is warranted.

## Supplementary Information

Supplementary Material 1.

## Data Availability

Data available on request from the authors.
